# Thematic Analysis of Challenges of Care Coordination for Underinsured and Uninsured Cancer Survivors With Chronic Conditions

**DOI:** 10.1001/jamanetworkopen.2021.19080

**Published:** 2021-08-13

**Authors:** Bijal A. Balasubramanian, Robin T. Higashi, Serena A. Rodriguez, Navid Sadeghi, Noel O. Santini, Simon Craddock Lee

**Affiliations:** 1 University of Texas Health Science Center at Houston (UTHealth) School of Public Health, Dallas; 2 Harold C. Simmons Comprehensive Cancer Center, Dallas, Texas; 3 University of Texas Southwestern Medical Center, Dallas; 4 Parkland Health and Hospital System, Dallas, Texas

## Abstract

**Importance:**

Although a majority of underinsured and uninsured patients with cancer have multiple comorbidities, many lack consistent connections with a primary care team to manage chronic conditions during and after cancer treatment. This presents a major challenge to delivering high-quality comprehensive and coordinated care.

**Objective:**

To describe challenges and opportunities for coordinating care in an integrated safety-net system for patients with both cancer and other chronic conditions.

**Design, Setting, and Participants:**

This multimodal qualitative study was conducted from May 2016 to July 2019 at a county-funded, vertically integrated safety-net health system including ambulatory oncology, urgent care, primary care, and specialty care. Participants were 93 health system stakeholders (clinicians, leaders, clinical, and administrative staff) strategically and snowball sampled for semistructured interviews and observation during meetings and daily processes of care. Data collection and analysis were conducted iteratively using a grounded theory approach, followed by systematic thematic analysis to organize data, review, and interpret comprehensive findings. Data were analyzed from March 2019 to March 2020.

**Main Outcomes and Measures:**

Multilevel factors associated with experiences of coordinating care for patients with cancer and chronic conditions among oncology and primary care stakeholders.

**Results:**

Among interviews and observation of 93 health system stakeholders, system-level factors identified as being associated with care coordination included challenges to accessing primary care, lack of communication between oncology and primary care clinicians, and leadership awareness of care coordination challenges. Clinician-level factors included unclear role delineation and lack of clinician knowledge and preparedness to manage the effects of cancer and chronic conditions.

**Conclusions and Relevance:**

Primary care may play a critical role in delivering coordinated care for patients with cancer and chronic diseases. This study’s findings suggest a need for care delivery strategies that bridge oncology and primary care by enhancing communication, better delineating roles and responsibilities across care teams, and improving clinician knowledge and preparedness to care for patients with cancer and chronic conditions. Expanding timely access to primary care is also key, albeit challenging in resource-limited safety-net settings.

## Key Points

**Question:**

What are the barriers to coordinating care for underinsured and uninsured patients with cancer and chronic conditions during and after cancer treatment at a safety-net health system?

**Findings:**

This qualitative study of interviews and observation of 93 health system stakeholders identified system-level challenges (ie, challenges to accessing primary care, lack of communication between oncology and primary care clinicians, leadership awareness of care coordination challenges) and clinician-level challenges (ie, unclear role delineation, lack of clinician knowledge and preparedness to manage cancer and chronic conditions).

**Meaning:**

These findings suggest that survivorship care delivery strategies that bridge oncology and primary care may be required to meet the care coordination needs of underinsured and uninsured patients with cancer amid the fragmented US health care system.

## Teaser

This qualitative study assesses barriers to coordinating care for patients with cancer and chronic conditions during and after cancer treatment.

## Introduction

Care delivery for cancer survivors with cooccurring chronic conditions^1^ is changing rapidly. Countervailing forces of clinical complexity, payment reform, and health information technology innovations have increased expectations for efficiency and effectiveness.^2^ Movement toward value-based care by the Centers for Medicare & Medicaid Services and other payers increasingly holds clinicians accountable through guideline-based bundled payments^3,4^ and risk-based reimbursement, with substantial implications for optimal care of noncancer comorbidities such as diabetes and hypertension.^5,6^ As more clinicians become involved in cancer treatment, surveillance, and survivorship care, communication across multiple clinical teams becomes increasingly difficult, and care coordination can be jeopardized.^7,8^

Models for comprehensive management of patients with cancer and comorbidities are still evolving.^9,10,11^ To date, most cancer care management efforts have focused within oncology on increasing coordination between cancer treatment modalities (ie, chemotherapy, radiation, and surgery).^12,13,14^ Efforts to develop and test new survivorship care delivery models have been recommended for the growing number of cancer patients with multiple chronic conditions for whom coordination is especially lacking.^15,16,17^

Although care coordination is a universal problem, it is critical to address barriers for underinsured and uninsured patients coping with competing demands, increased medical complexity, and substantial social and economic barriers who often seek care in safety-net systems.^18,19,20^ Few studies have focused on delineating system factors related to delivering coordinated care to underinsured and uninsured cancer survivors. To address this gap, we describe challenges and opportunities for improving care for cancer survivors with chronic conditions. In this study, cancer survivors with chronic conditions were patients who have preexisting chronic conditions (diabetes, hypertension, heart disease, chronic obstructive pulmonary disease, or chronic kidney disease), were diagnosed with stage I, II, or III breast or colorectal cancer, and have completed cancer treatment (consisting of chemotherapy, radiation, and/or surgery).^21^

## Methods

### Design

We conducted a multimodal qualitative study in conjunction with a prospective quasi-experimental pragmatic trial.^22^ Data were collected as part of a baseline assessment of the health system before implementing a care coordination intervention among patients with stage I, II, or III breast or colorectal cancer with chronic conditions (diabetes, hypertension, heart disease, chronic kidney disease, or pulmonary disease).

### Setting and Study Participants

Parkland Health and Hospital System is a public safety-net system serving underinsured Dallas County, Texas residents that includes primary care, specialty care, an ambulatory surgery center, and inpatient hospital facility.^23^ Clinicians and staff used a common electronic health record system (Epic) that included specialty-specific modules (such as Beacon for oncology). Patients accessed primary care through a network of 13 clinics located in predominantly low-income Dallas County communities. Ambulatory oncology service was centrally located on the Parkland main campus adjacent to the tertiary care hospital to facilitate multidisciplinary cancer care. In 2016, more than 80% of Parkland’s patients with breast cancer or colorectal cancer had hypertension, and more than one-third had diabetes (Parkland Cancer Registry). Patients diagnosed with cancer could be referred to oncology by their primary care clinician, by the hospital, or from an outside facility.

Study participants included health system stakeholders: hospital leadership, oncology and primary care clinicians, clinical staff (nurses, patient navigators, care coordinators) and nonclinical staff (schedulers, financial assistants) (Table). We began with a purposive sampling approach with serial chain referral to include key participants from specialty care and urgent care as they were identified by oncology and primary care stakeholders. We did not solicit gender, race/ethnicity, or other demographic data from health system stakeholder participants because these data were not germane to the research question.

**Table.** Qualitative Data Collection

Purpose	Unique persons, No.	Encounters, No.^a^
Leadership engagement		
Primary care leaders	14	4
Parkland executives	5	1
Acute Response Care leadership	2	1
Oncology practice leads	9	11
Diabetes leadership	1	1
Subtotal	31	18
Primary care		
Administrators (site administrator, business manager)	4	4
Clinicians (including lead MD)	13	13
Nurses (including nurse manager)	2	3
Social workers and case managers	4	5
Acute Response Clinic clinicians and staff	4	3
Subtotal	27	28
Oncology outpatient care		
Advanced-practice clinicians (ie, NP, PA)	5	19
Physicians	3	6
Nurses	5	5
Social worker	1	2
Administrative staff (eg, patient onboarding)	3	4
Oncology practice leads	2	6
Subtotal	19	42
Chronic condition management		
Geriatrics or HIV clinician	1	1
Diabetes or hypertension specialty group	3	2
Diabetes clinicians and educator	3	2
Surgery C (colorectal cancers) clinician and staff	2	3
Subtotal	9	8
EHR, IT, or registry		
IT professionals	3	2
Parkland Cancer Registry staff	1	2
Subtotal	4	4
Other		
Breast center manager (newly diagnosed patient referrals)	1	1
Parkland Financial Services staff	1	1
Parkland Health Innovations staff	1	1
Subtotal	3	3
Total	93	103

Abbreviations: EHR, electronic health record; IT, information technology; NP, nurse practitioner; PA, physician assistant.

^a^ Some participants engaged over multiple encounters.

### Data Collection

Data collection occurred from study start in May 2016 to intervention start in July 2019. Researchers conducted semistructured interviews and observations of work processes and meetings to delineate care delivery and identify gaps in care coordination. Data collection began in ambulatory oncology, expanding into associated practice sites relevant to chronic condition management. Some participants were engaged multiple times for clarification of ideas (Table). This resulted in interview transcripts, researchers’ observation and meetings fieldnotes, and documents (eg, ambulatory specialty and primary care procedures). To ensure rigor we followed the Consolidated Criteria for Reporting Qualitative Research (COREQ) reporting guideline.^24^ Informed verbal consent was obtained from all study participants in accordance with the protocol approved by the University of Texas Southwestern Medical Center institutional review board and site approval by Parkland Health and Hospital System Office of Research Administration.

### Data Analysis

Data collection and analysis occurred iteratively allowing for real-time data interpretation to identify patterns, organize findings by service location (eg, primary care, oncology) and theme (eg, communication, process barriers), verify accuracy of findings, and obtain additional information about areas of nonconvergence.^25^ Three qualitatively trained team members (including R.T.H. and S.C.L.) developed a deductively driven thematic codebook based on a preliminary review of transcripts, fieldnotes, and documents. The team triple-coded the first 12% of transcripts using this preliminary codebook, meeting to resolve coding discrepancies by consensus, add emergent themes, refine, and finalize the codebook. Four team members (including R.T.H.) double-coded remaining data in a matrix pattern to ensure that coders were paired evenly across transcripts for balanced analysis using the finalized codebook. Analysts then summarized findings from thematically coded data in reports synthesizing major findings and identified representative quotes and excerpts. All members of the research team participated in selecting themes from these synthesized reports for this manuscript. Data collection ended when thematic saturation was reached as determined by team consensus. Data were analyzed from March 2019 to March 2020.

## Results

After interviewing and observing 93 health system stakeholders included in this qualitative study, we described opportunities and challenges to coordinate care delivery for cancer survivors with chronic conditions at the system and clinician level. We defined the term survivors as patients diagnosed with stage I, II, or III cancer who have completed cancer treatment (consisting of chemotherapy, radiation, and/or surgery) and who have entered the surveillance stage of the cancer control continuum.^21^ The Figure depicts expected levels of patient engagement with care clinicians and places in the cancer control continuum we observed system- and clinician-level gaps in care coordination.

**Figure.**
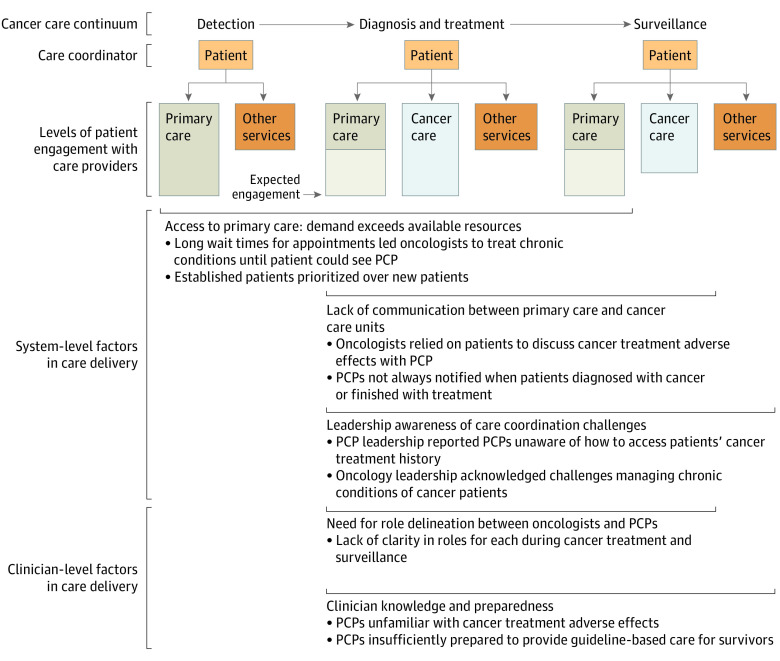
Gaps in Care Coordination for Patients With Cancer and Chronic Conditions Primary care included chronic disease care, including specialty care (eg, diabetes wound care, cardiology). Other services included ancillary services (eg, social work, laboratory, pharmacy, financial services, smoking cessation). Cancer care included medical, surgical procedures, and radiation oncology. PCP indicates primary care practitioner.

### Health System–Level Factors

Key health system–level factors associated with care coordination among cancer survivors with chronic conditions were found. These included challenges accessing primary care, lack of communication between oncology and primary care practitioners (PCPs), and leadership awareness of care coordination challenges.

#### Challenges Accessing Primary Care and Its Consequences

Many patients with cancer had cooccurring diabetes or hypertension, and oncology nurses routinely referred patients to primary care. However, primary care clinics faced enormous demand, often resulting in scheduling backlogs for existing patients and long delays for new patients. Wait times greater than 6 months were common. We did not observe systematic follow-up to ensure patients completed appointments.

“If everybody could get a PCP that would be fabulous, but what happens is I’ll refer them, then they’ll come back 6 months later and say, well I called but I’m still on the waiting list,” said an oncology clinician.

“These patients don’t just have cancer. They have 900 other issues that can make their cancer more complicated to treat, their surgery more difficult to perform … If they could get some of those issues treated, then our jobs would be a lot easier,” said an oncology clinician.

Patients with an established PCP faced fewer challenges accessing primary care because existing patient appointments were prioritized over new patient appointments. However, even short delays for primary care referrals shifted patients to emergency or urgent care facilities for chronic condition management.

“Routine care gets pushed aside. They use the emergency room because they can’t get in to the PCPs,” said an oncology clinician.

“They need hypertension management, all this stuff they’re not getting. So then they’re all out of their medicine, I can’t get them in [to a PCP], so I end up sending them to urgent care just to get their refills,” said an oncology clinician.

Given challenges accessing appointments in primary care, oncologists were faced at times with decisions about managing patients’ chronic diseases. Some oncologists knowingly performed primary care tasks because it seemed expedient for a patient’s care.

“They want us to be their PCP … I have 15 minutes to see my postop patient and she wants to show me her bad tooth. What do you do? Are you going to send her home with a bad tooth? You write for some antibiotics. You call up the coordinator for oral surgery and you try to get her in. Now your 15-minute postop appointment turned into an hour dealing with this patient with a bad tooth,” said an oncology clinician.

“A lot of times I treat the patient for high blood pressure rather than waiting … And at least that way, while I’m waiting for the referral, I don’t have to worry that his blood pressure’s out of control,” said an oncology clinician.

Oncologists’ responses to managing diabetes or hypertension varied depending on whether they judged a condition as interfering with ongoing cancer treatment and with their comfort level managing chronic conditions. Most oncologists temporarily managed acute presentation of chronic conditions in order to proceed with cancer treatment.

“We’re not going to halt treatment while the patient gets normalized blood pressure … maybe the oncologist treats the hypertension. I think a lot of times they do, but it’s all depending on what the doctor wants to do,” said an oncology clinician.

“I’m not going to hold treatment, especially when it’s chemo … In my experience, what happens is they go ahead and give the chemo, make the referral to primary … and then that whole time, his hypertension is not treated adequately,” said an oncology clinician.

#### Lack of Communication Between Oncologists and PCPs

Both oncologists and PCPs felt communication between them was lacking. Oncologists often relied on patients to communicate about cancer treatment side effects to PCPs. However, patients’ lack of consistent contact with PCPs could result in substantial consequences because some cancer medicines exacerbate chronic diseases.

“Primary care physicians might not be very conversant to the side effects of the oncology medications…are these medications affecting blood glucose? Are they on steroids? If the patient has neuropathy, is it due to the diabetes or the medications?” said a PCP/endocrinology clinician.

PCPs wanted more direct communication from oncologists about how to manage patients’ cancer-related primary care needs, especially when patients completed cancer treatment. “When they are discharged from oncology, there should be some type of communication with the PCP…so we can just keep an eye on [patients],” said a PCP.

PCPs also wanted to be notified when their patients were diagnosed with cancer. “Currently, there is no system in place ... There is no coordination. We have so many patients, we don’t really know if they are diagnosed until we get something sent by specialty clinics, or they come in, or they call, or they end up in the emergency room,” said a PCP.

#### Leadership Awareness of Care Coordination Challenges

Primary care and oncology leaders acknowledged significant challenges to coordinating care for cancer survivors with chronic conditions, suggesting the potential for adverse health outcomes. Oncology leaders reported extensive challenges meeting patients’ chronic disease needs during cancer treatment. One primary care leader described a pattern of lack of communication between primary care clinicians and oncologists once a patient is diagnosed with cancer.

“When a patient is being seen in the primary care service and is diagnosed with cancer ... we kind of lose that patient. Some patients continue coming to our primary care services for follow-ups, but many patients just start going to the oncology service and then we stop seeing them for a year or two or whatever … We should be following these patients together, not just after they complete their treatment,” said a primary care administrator.

### Clinician-Level Factors

Several clinician-level factors were associated with coordinated care delivery. These included a need for role delineation between oncology and primary care clinicians and lack of clinician knowledge and preparedness to manage the effects of cancer and chronic conditions.

#### Role Delineation

Oncologists and PCPs sometimes differed in their understanding of who was responsible for performing cancer surveillance testing (eg, a mammogram for a survivor of breast cancer) and whether responsibility shifted over time. For example, while most agreed that survivors should be managed by oncologists during the first 5 years, they disagreed about whether oncologists or PCPs should order tests for a survivor at 10 or more years.

“Who is supposed to follow that cancer follow-up frequency? Is Oncology gonna do it or Primary Care has to do it? So if they can put ‘we continue to follow’ [in the EMR], or if the patient is discharged from Oncology and Primary Care has to follow up or whatever, then make that really more clear,” said a PCP.

“The ideal thing would be a survivorship program to see all survivors, at all stages. But [the Medical Oncology Clinic] doesn’t have the bandwidth to take all survivors now,” said an oncology clinician.

One PCP felt it might be better for cancer surveillance testing to continue to be performed by oncologists because some patients fall out of primary care during and after a cancer diagnosis but continue to follow up with their oncology team. “Cancer is not like any other disease. We really have to be very careful. If you leave [cancer surveillance] with the PCP … [patients] might not show up ... I don’t want patients to fall through the cracks,” said a PCP.

Several PCPs felt unclear about their role in managing pain medications, and many were uncomfortable prescribing medications for cancer-related pain. “Cancer patients with complex pain medications—I believe this should be the oncologists that should be handling. They should feel comfortable prescribing all these medications, not just talking to us, because they see these patients all the time ... We have to agree on how this should be handled,” said a PCP.

#### Lack of Clinician Knowledge and Preparedness

For some primary care clinicians, lack of familiarity with cancer treatment effects contributed to their discomfort managing care for cancer survivors. “I have a patient with [brain metastases]…and he was started on 3 seizure medications, and he doesn’t have seizures but I guess they have a role in treating headaches. He’s on 3 medications that I am not familiar with. The last 3/4 months the medication refill comes to me ... I am uncomfortable with these 3 medications. I don’t prescribe 3 seizure medications to treat a headache,” said a PCP.

Primary care clinicians also felt they were insufficiently prepared to provide guideline-based care for cancer survivors. “Those patients who are discharged from the oncology clinic ... what type of surveillance do we need to do? ... What are the guidelines to monitor those types of patients?” said a PCP.

## Discussion

We identified substantial barriers at the system- and clinician-levels to delivering coordinated care to cancer survivors with chronic conditions at an integrated safety-net health system. We found that patients with cancer and chronic conditions lacked consistent and timely connection to primary care, especially when they were not already established with a PCP. Consistent with other studies, even in integrated care settings, the “siloed functioning of health systems” hinders fluid transitions between oncology and primary care for patients with cancer and comorbidities.^26^ Given a lack of effective care coordination, patients became de facto coordinators of their own care.^26,27^

When patients did access primary care, we found a disconnect between perceived roles and responsibilities of primary care and oncology clinicians. This was compounded by lack of communication about critical aspects of patients’ cancer diagnoses, cancer treatments, and chronic disease needs. Prior studies among independent physicians previously described this disconnect.^8,28,29^ A recent study of 12 advanced primary care practices identified the absence of a recognized, distinct clinical category of survivorship in primary care, a lack of actionable information to treat this patient population, and current information systems unable to support survivorship care as additional challenges.^30^ One would expect less confusion in roles and responsibilities among physicians of an integrated system,^31^ such as Parkland, where clinical teams share overarching organizational processes and policies.^26^ At the time of this study, Parkland did not have routinized dissemination of treatment summary and survivorship care plans from oncology to primary care. Access within the EHR to oncology-specific records by primary care still needs to be addressed. As noted, modular design of EHR functionality creates additional barriers to primary care clinician teams accessing information from their oncology counterparts.^32,33^ Our findings suggest this may contribute to lack of clinician knowledge and preparedness to manage the effects of cancer and chronic conditions, thereby exacerbating challenges in delineating roles and responsibilities for survivorship care.

Analyses of Centers for Medicare & Medicaid Services claims data indicate that patients with cancer seen by both primary care and oncology clinicians were more likely to receive preventive care and appropriate chronic disease care.^34,35,36^ With increasing numbers of survivors, delivering coordinated care for cancer and chronic diseases requires explicit inclusion of primary care in their care. Although current oncology practice has successfully integrated multidisciplinary cancer care, it has not yet systematically incorporated primary care into this multidisciplinary care model. In contrast, the disconnect between oncology and primary care pushes the burden of coordination from the health system to the patient as the central “repository of information,”^15,37^ contributing to suboptimal outcomes. Our study findings highlighted this disconnect during cancer treatment through the survivorship phase. Health system leaders will need to work across clinical disciplines to enhance primary care capacity to care for survivors and support oncology in implementing shared care approaches with primary care.

We found primary care, oncology, and other clinical disciplines were interacting with each other in the care of patients with cancer and chronic conditions, but with substantial barriers (Figure). To overcome these barriers, primary care and oncology leaders may need to create shared mental models, improve communication about care, and leverage health information technology. Primary care, as a discipline, will also need to enhance training on cancer survivorship in primary care to meet the demand of the growing population of cancer survivors.^30^

### Strengths and Limitations

This analysis was strengthened by its grounded theory approach. Iterative data collection and analysis facilitated both interpretation and development of probes. Another strength was our focus on a vertically integrated safety-net health system as a strategic exemplar to elucidate challenges of caring for underinsured and uninsured patients with cancer and chronic conditions. Unlike other integrated systems, such as Kaiser Permanente, Parkland’s patient population is disproportionately uninsured, with almost one-third funded through charity programs, and another one-quarter enrolled in Medicaid.^23^ Among patients with stage I, II, or III breast or colorectal cancer on Parkland’s cancer registry, more than 60 percent were uninsured. As a county safety-net, the system had comparatively limited resources, overextended clinicians and staff owing to exceptionally high patient volume and patients with medically complex conditions with multiple unmet needs related to social determinants of health. However, the integrated nature of the health system facilitated in-depth data collection from multiple stakeholders across different clinical specialties.

Our study’s focus also had limitations. We identified care coordination challenges among patients who were part of a system of primary care clinics owned and operated under the same governance as the tertiary care hospital and specialty care services, including oncology. However, for patients with cancer who access primary care in independent community settings such as federally qualified health centers but access specialty care through separate organizations, staying connected or establishing new connections with primary care may pose an even more substantial barrier to receiving comprehensive, coordinated care for both cancer and chronic disease.^38,39^ The absence of direct pathways between primary and specialty care in independently owned, virtually integrated settings (linked only by contractual agreement)^31^ is likely more complicated where documentation and communication involves independent EHRs. Research with independent primary care clinics with external oncology practice partners have found similar challenges and will require appropriately adapted interventions.^30^

## Conclusions

This qualitative study identified multilevel challenges associated with disrupted care coordination. These findings suggest that, given interdependencies between levels within a single health system, awareness and engagement by system leadership may be needed to create the conditions necessary to actualize and sustain communication across teams to support comprehensive cancer survivorship care.^16^ One immediate target is closed-loop communication, ensuring both vertical and horizontal communication pathways.^40^ Health information technology tools may also foster team effectiveness by better identifying high-risk patients, monitoring outcomes, and managing task interdependence during transitions in care.^41,42,43^

Care coordination for underinsured and uninsured cancer survivors with comorbidities will continue to be an important issue given changing US demographics, early detection, and advances in cancer therapies that have extended cancer survivorship. Without robust communication systems and care pathways between primary care and oncology, such patients are at risk of increasingly poor outcomes, exacerbated by the patchwork nature of the US health care system. Closed-loop communication, role clarification, leadership support, and health information technology improvements to comanage patients during care transitions may provide a start toward enhanced team-based care.
